# Early-life microbiome trajectories as biomarkers to predict health outcomes

**DOI:** 10.20517/mrr.2026.03

**Published:** 2026-06-23

**Authors:** Raphaela Joos, Aonghus Lavelle, Eugene Dempsey, Catherine Stanton, R. Paul Ross

**Affiliations:** ^1^APC Microbiome Ireland, University College Cork, Cork VGR3+XF, Ireland.; ^2^School of Microbiology, University College Cork, Cork T12 Y337, Ireland.; ^3^Department of Anatomy and Neuroscience, University College Cork, Cork VGV2+82, Ireland.; ^4^Department of Paediatrics and Child Health, University College Cork, Cork T12 DC4A, Ireland.; ^5^INFANT Irish Centre for Maternal and Child Health Research, University College Cork, Cork VFMP+MJ, Ireland.; ^6^Teagasc Food Research Centre, Fermoy P61 C996, Ireland.

**Keywords:** Early-life microbiome, infant development, machine learning, healthy trajectories, microbial succession, paediatric health

## Abstract

The early-life gut microbiome is tightly linked to different aspects of infant development. Microbial colonisation patterns have been repeatedly shown to play a role in a variety of paediatric outcomes, ranging from metabolism and immune function to neurodevelopment. Concomitantly, the identification of early-life biomarkers is crucial, especially considering that for various conditions, reliable diagnostic tools only emerge in early childhood. As such, microbiome data collected in the first two years of life may offer valuable prospects for early detection, prevention, quantification or even correction of adverse health trajectories. With the increasing availability of high-resolution microbiome data, researchers are leveraging both traditional statistical approaches and machine learning (ML) methods to analyse the evolution of these complex microbial communities. While statistical models are well-suited for identifying associations between microbiome features and health states, ML methods allow for predicting health outcomes from those features. This review explores the role of the early-life gut microbiome in infant health and development, with a focus on how data acquisition and analytical methods can shape current knowledge. We contrast statistical approaches with ML methods, summarising key findings on microbial succession and factors influencing it. By addressing current challenges and identifying areas for methodological refinement, we aim to discuss the potential of the microbiome in the assessment of current and future health states of an individual and aid in the development of more robust, clinically-relevant models for paediatric care.

## INTRODUCTION

The human gut microbiome - a complex microbial community, its gene collection and metabolic potential - adapts dynamically across a lifetime, yet a critical period is the initial assembly in early life^[1-3]^. It is initiated from birth onwards, with differential intrinsic and external exposures, including birth mode, feeding patterns and lifestyle factors shaping the colonisation trajectory^[4-8]^. Mounting evidence suggests that these early microbial communities not only correlate with health conditions in infancy but may also distinguish between divergent health trajectories - current and future^[9-14]^. While the former findings rely on statistical analysis, which remains indispensable for detecting relationships between microbial communities and host phenotypes, the latter are obtained by employing machine learning (ML), which extends analytical capacity from association toward prediction. ML is a branch of artificial intelligence capable of parsing complex data such as microbial metagenomes for meaningful patterns^[15]^. Indeed, ML algorithms have been found successful in detecting microbiome-host interactions, predicting host phenotypes as well as potential disease risks based on microbial communities^[16-18]^. It thereby serves as a tool for translating microbiome data into feasible clinical interventions used for potential microbial biomarker identification.

Several obstacles remain in employing operational microbiome-based predictive models in clinical practice. High inter-individual variability^[19]^, driven by genetic, environmental, and lifestyle factors^[20,21]^, has so far prevented the establishment of a benchmark ‘healthy’ microbiome^[22]^. Without such reference points, defining optimal community structures or standardising risk prediction remains difficult. Moreover, while ML can identify prediction patterns linking microbial profiles to health outcomes, prediction does not equate to causality^[23]^, and the opaque nature of many algorithms raises concerns about interpretability^[24]^.

To date, most existing reviews in the field have focused on either microbiome-health associations in early life^[25-29]^, or on machine learning applications in adult populations^[16,23,24]^. Given the central role of early development in shaping long-term health trajectories, and the limited effectiveness of many diagnostic tools in later childhood^[30,31]^, microbiome-based prediction in infancy holds considerable potential for earlier risk stratification, personalised intervention, and disease prevention. However, the early-life microbiome presents distinct analytical challenges, including rapid temporal dynamics, strong environmental sensitivity, and high inter-individual variability. These considerations in mind, a structured overview of how machine learning models perform in early-life cohorts, and how both methodological and biological factors influence predictive outcomes, is currently lacking.

This review addresses this gap by providing a focused evaluation of machine learning applications in the infant gut microbiome, with an emphasis on predictive modelling from birth to two years of age. In contrast to previous reviews, we critically assess how predictive models are constructed, which outcomes they target, and how performance varies across study designs and analytical frameworks. We further integrate key methodological aspects of microbiome research, including sequencing strategies and pre-processing pipelines, to evaluate their impact on model performance and cross-study comparability. By linking early-life microbial ecology with analytical and computational considerations, this review provides a structured framework for interpreting existing predictive evidence and clarifies the current limitations and requirements for developing robust, generalisable, and clinically meaningful microbiome-based prediction in infancy.

## DATA ACQUISITION, PROCESSING AND ANALYSIS IN MICROBIOME RESEARCH

The initial stages of data acquisition and handling are essential for subsequent data quality and analysis. Microbial community profiling typically progresses from sample collection (e.g. saliva, stool or skin swabs) to DNA extraction, sequencing, preprocessing and data analysis. DNA extraction involves breaking open microbial cells using chemical, enzymatic, or mechanical methods to release DNA, followed by its purification from contaminants^[32]^. Importantly, microbiome data are highly sensitive to variation in these pre-analytical steps. For example, DNA yield varies across stabilisation methods, time to freezing, freeze-thaw cycles, and storage time^[33-37]^. In addition, DNA recovery can vary substantially based on extraction protocols, particularly due to lysis strategies and efficiency, which disproportionately affects Gram-positive bacteria with more resilient cell walls^[38-41]^.

Such differences in sample handling can introduce significant bias not only to DNA yield but also to inferred microbial profiles, which is especially critical in infant samples, considering their limited microbial community size^[37,38,42]^. Sequencing requires the preparation of DNA libraries, by either amplifying specific marker regions or sequencing all genetic material^[43]^. The two main approaches are whole-metagenomic shotgun sequencing (WGS) and 16S rRNA (16S) gene sequencing. The 16S gene, a genetic marker present in almost all bacteria, contains both conserved and variable regions, allowing taxonomic identification of bacteria and archaea typically up to the genus level, but without functional insight^[43-45]^. For WGS, the genetic material found in samples is sliced into smaller parts (i.e. as if being shot with a gun), and all DNA is sequenced, thereby allowing sequencing of all microbes, including bacteria, archaea, fungi and viruses^[43,44]^. While WGS enables higher taxonomic resolution and functional analysis, it is more computationally demanding, prone to host contamination and relies on less complete reference databases^[20,43,44,46,47]^. Although decreasing cost makes WGS more accessible^[44]^, both approaches present challenges in sequence alignment and downstream interpretations.

The consequent processing and analysis of such complex and large data is challenging, both in terms of aligning the genetic sequences with reference databases and investigating associations between genetic material and disease phenotypes. While bioinformatic pipelines dealing with the former are extensively reviewed elsewhere^[20,46]^, we will focus on summarising challenges around the downstream analysis of microbiome data and attempts to combat them. Microbial communities underly principles of biological ecology, manifesting in complex relationships between microbes, and demanding consideration of such relationships in analysis^[22]^. Moreover, interpersonal variability of compositional microbial profiles based on subjective microbial exposure makes for dataframes with large amounts of columns and zeros, i.e. sparse yet skewed feature distributions, which increases with larger sample sizes^[20,43,48]^. Equally, large amounts of confounding variables ranging from technical considerations such as read counts or sequencing batches, study design factors including repeated measures, as well as covariates such as lifestyle and diet, further complicate microbiome data analysis^[48]^.

**Figure 1:**
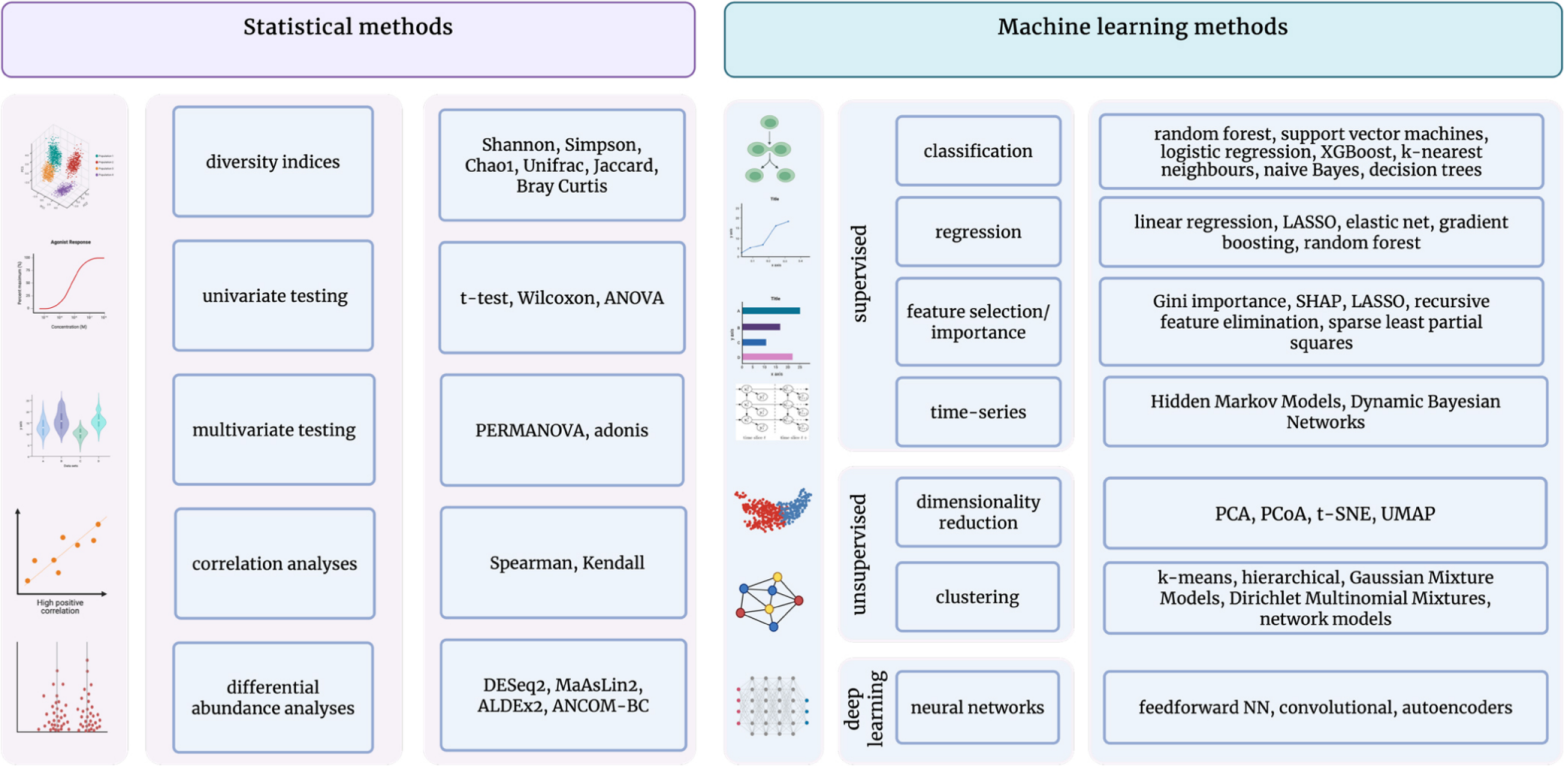
Downstream data analysis in microbiome research; Conceptual schematic summarizing commonly used statistical and machine-learning approaches for the analysis of microbiome data, highlighting methods for quantifying microbial features, modeling community structure, and extracting biologically meaningful insights. Created in BioRender. Lavelle, A. (2026) https://BioRender.com/qwizjk8 ANOVA: Analysis of variance; PERMANOVA: permutational multivariate analysis of variance; SHAP: SHapley Additive exPlanations; LASSO: Least Absolute Shrinkage and Selection Operator; PCA: principal component analysis; PCoA: principal coordinates analysis; t-SNE: t-distributed stochastic neighbor embedding; UMAP, uniform manifold approximation and projection; NN: neural networks.

As such, a key feature of microbiome data analysis is to narrow down features, which also improves the interpretability of final outputs^[23]^. These challenges are currently being tackled by uniquely creative solutions, which can broadly be divided into two categories, as shown in Figure 1: statistical and ML approaches. Statistical methodology aims to use exploratory approaches to answer specific hypotheses, most of which focus on microbiome differences at different developmental stages and between health and disease conditions. As such, statistical methods assess differences between individuals or groups in microbial community diversity or abundances of specific taxa, including compositional features or functional pathways. For the latter, referred to as differential abundance analysis, tests such as the Wilcoxon rank sum test or Fisher’s test, as well as a variety of software tools including ANCOM-BC2^[49]^, DESeq2^[50]^, or MaAsLin2^[51]^ are used, employing varying approaches such as mixed linear or binomial models. Lastly, taxa abundances can be correlated with any given clinical feature, providing a measure of the relationship between them. Taken together, statistical approaches provide interpretable differences between samples, quantifying group-level differences and indicating a degree of relatedness.

ML approaches, in turn, are able to handle greater amounts of more complex data, parsing it for underlying patterns and allowing statements regarding its predictive qualities. They are considered a type of artificial intelligence due to their intrinsic capacity to conduct trial and error processes, adjusting to outcomes automatically^[15]^. Within ML, models can be categorised as supervised or unsupervised based on whether outcomes or predictors are set a priori^[15]^. Unsupervised ML methods are predominantly used for dimensionality (i.e. feature) reduction, as well as clustering approaches such as k-means or hierarchical clustering^[16,23,24]^. The goal of both is to identify how samples or features naturally group together, revealing underlying patterns or structures in the microbiome without prior knowledge or outcome labels.

Supervised ML methods in turn leverage a predefined set of factors to predict a specific continuous (regressor models) or categorical (classification models) outcome, making them a valuable tool for identifying clinical biomarkers or diagnosis^[15,23,48]^. The respective workflow consists of several steps, starting with feature selection. Besides selecting the outcome variable of interest, a variety of dependent variables are tested for their predictive qualities, a process referred to as the training phase^[23]^. This process is followed by a testing phase, where the selected features are presented with unseen data, resulting in a variety of model performance metrics^[23,43]^. Subsequently, the model can be improved by an iterative flow through the previous steps, altering the selected features or model parameters to optimise predictive quality^[23]^.

Taken together, both statistical and ML approaches are essential and complementary in microbiome data analysis. While statistical methods are crucial in data preparation and characterisation, ML methodology allows for the recognition of patterns, clusters or predictive qualities of high-dimensional and non-linear microbiome data. As such, and especially in the light of the infant microbiome, ML presents an exciting avenue to extend analytical insight from association toward prediction of present and future health states. While prediction does not imply causation, integrating ML with longitudinal data and experimental validation strategies can help to move further towards causal relationships between microbial profiles and diseases.

## THE EARLY-LIFE MICROBIOME: MICROBIAL SUCCESSION AND ITS ROLE IN INFANT DEVELOPMENT

The microbial ecosystem in the infant gastrointestinal tract begins to establish itself through a process referred to as microbial succession, whereby different species colonise the body in a relatively ordered yet individualised sequence, gradually building a stable and complex microbial community^[1,13,19,52,53]^. Universally, infants experience initial colonisation at birth^[54]^, and the transmission process is thought to happen through both genetic predisposition and direct microbial exposure^[55]^. As such, microbial colonisation is directed by perinatal factors such as individual gestational age and mode of delivery, reflected in vaginal births seeding microbes from the maternal vaginal and gastrointestinal tract, while infants born via cesarean section are primarily exposed to skin- and hospital-associated microbes^[1,4,56]^. Accordingly, community structures of the two groups differ robustly in the first few months of infancy, with vaginally-born infants displaying higher numbers of species belonging to *Lactobacillus, Bacteroides* and *Bifidobacterium*, while infants born via cesarean section harbour higher amounts of *Staphylococcus, Veillonella* and *Streptococcus* species^[1,19,57]^.

Similarly, various factors, including antibiotic exposure, environmental microbial contact, and maternal health, as well as early-life feeding patterns, play key roles in the individuality of succession^[1,19,58]^. While in general, microbial profiles of exclusively milk-fed infants are dominated by aerobic and facultative anaerobic taxa of Bifidobacteriaceae and Enterobacteriaceae^[8,52,59,60]^, individual differences based on varying amounts of either breastmilk or formula persist^[1,6,13,57,61-64]^. Such dissimilarities are thought to trace back to breastmilk containing immunoregulatory compounds^[65,66]^ and promoting more limited amounts of species metabolising high amounts of prebiotic human milk oligosaccharides (HMOs) such as *Bifidobacterium longum* subsp*. infantis*^[67]^, while formula fosters a more diverse but less specialised microbiota^[8,19,62,68-71]^. The introduction of solid foods, respectively, the stepwise cessation of breastmilk from around six months of age, encourages a major microbial transition^[1,8,13,19,72]^. As a result, the presence of more anaerobic taxa capable of metabolising complex carbohydrates and proteins, such as Bacillota (formerly Firmicutes), Bacteroidota (formerly Bacteroidetes), and Clostridiales increases^[1,13,73]^. Continuing throughout toddlerhood, higher abundances of species such as *Faecalibacterium prausnitzii* and *Methanobrevibacter smithii*, as well as increasing diversity and complexity of microbial community, result in mature, adult-like microbiota typically by age three years^[1,19,55,74-77]^. As such, microbial succession follows predictable phases over the first two years of life, despite the various individual external and internal factors influencing the trajectory^[19,53,74]^.

Building on the understanding of microbial succession in early life, researchers have increasingly investigated how these dynamic microbial patterns relate to infant health outcomes. In most cases, such a role is established by statistically identifying factors associated with an outcome, followed by demonstrating differences in microbial profiles based on the same factors and the outcome, drawing an interactive effect triangle. As such, the early-life microbiome is thought to play a major role in healthy development and associated health and disease conditions, including, among others immuno-mediated diseases such as type 1 diabetes, inflammatory bowel disease, asthma, atopic dermatitis, and food allergies, as well as neurodevelopment, metabolic conditions, including obesity and gastrointestinal conditions such as necrotising enterocolitis (NEC)^[27-29,78,79]^. Revealing these associations between the early-life microbiome and various paediatric disease states has fuelled interest in discovering microbial signatures that could serve as predictive biomarkers.

## THE EARLY-LIFE MICROBIOME AS A PREDICTOR OF HEALTH

Early-life ML studies harness the predictive potential of the infant gut microbiome and can be classified into two broad categories, as summarised in Tables 1 and 2: predicting host phenotypes based on (groups of) individual microbial taxa or based on microbial maturation indices.

**Table 1:** Predictive studies using microbial profiles to predict infant phenotypes

**Year**	**Authors**	**Dataset**	**Condition**	**Age**	**Participants**	**Samples**	**Data**	**Model**	**Metric**	**Value**
2021	Boutin *et al.*^[96]^	[96]	Allergies	3-12 months & 5 years	343	545	16S/ITS2	RF	Accuracy	0.81
2022	Casaburi *et al.*^[88]^	[80,122-126]	Fecrotising enterocolitis	0-2 months	344	1,647	WGS	RF, GB	Accuracy	0.9
2024	Chen *et al.*^[127]^	[127]	Neonatal jaundice	0 days	196	196	16S	RF, GB, Lasso	AUC	0.91
2017	Dobbler *et al.*^[84]^	[84]	Necrotising enterocolitis	0-5 weeks	40	132	16S	RF	Accuracy	0.76
2021	Fernández-Edreira *et al.*^[92]^	[128]	T1 diabetes	0-2 years	33	124	16S	RF, SVM, GLM	AUC	0.8-0.98
2020	Hooven *et al.*^[85]^	[129]	Necrotising enterocolitis	0-2 months	161	2,895	16S	NN	AUC	0.23-0.9
2020	Korpela *et al.*^[98]^	[98]	Overweight	0-3 years	212	308	16S	RF	AUC	0.58-0.7
2022	Kortekangas *et al.*^[109]^	[109]	Environmental enteric dysfunction	6-30 months	610	1,569	16S	RF	Variance explained	0.245-0.277
2020	LeGoallec *et al.*^[77]^	[1,112,128,130]	Early-life exposures	2-36 months	300	1,570	WGS	RF, GB, EN, SVM, KNN, NB	AUC, R-squared & Mean Class Accuracies	0.63-0.81
2022	Lin *et al.*^[86]^	[89,129]	Necrotising enterocolitis	0-2 months	261	3595	16S/WGS	NN, SVM, LR	AUC	0.86-0.92
2022	Lou *et al.*^[106]^	[106]	Autism spectrum disorder	11 months-19 years	1,202	1,202	16S	RF	AUC	0.67-0.86
2020	Loughman *et al.*^[94]^	[94]	Colics, problem crying & behaviour	0-3 months & 2 years	118	236	16S	RF	AUC	0.65
2022	Lugo-Martinez *et al.*^[131]^	[131]	Growth faltering	0-1 month	357	2923	WGS	RF, LR, HMM, DMM	AUC	0.59-0.76
2025	Ma *et al.*^[91]^	[90]	Atopic dermatitis	0-3 years	112	112	16S	RF, GB, SVM, LR	AUC	0.83-0.98
2022	Martin *et al.*^[132]^	[132]	Allergic proctocolitis	1 week-1 year	160	954	16S	RF	Accuracy	0.76
2021	Masi *et al.*^[87]^	[87]	Necrotising enterocolitis	0-4 months	48	644	WGS	SVM, RF,	AUC	0.88-0.95
2016	McGeachie *et al.*^[133]^	[134]	Preterm	0-2 months	58	922	16S	DNB	Mean absolute error	0.01-0.14
2019	Metwally *et al.*^[10]^	[10]	Food allergy	0-3 years	148	658	WGS	HMM, NN, SVM, RF, LASSO,	AUC	0.44-0.69
2025	Naspolini *et al.*^[57]^	[57]	Feeding	3-9 months	525	966	WGS	RF	Accuracy	0.57-0.74
2021	Nguyen *et al.*^[135]^	[64]	Functional microbial capacity	6 weeks-1 year	375	440	16S	RF, EN, SVM, SPLS	R-squared	-0.056-0.118
2019	Olm *et al.*^[89]^	[89]	Necrotising enterocolitis	0-2 months	160	1,163	WGS	GB, RF, SMOTE	Accuracy	0.64
2022	Pärnänen *et al.*^[81]^	[81]	Feeding & antimicrobial resistance	1 month	46	46	16S	RF	Feature importance	
2025	Piteková*et al.*^[114]^	[114]	Febrile urinary tract infection	0-17 years	35	35	16S	RF	AUC	0.87
2018	Rahman *et al.*^[80]^	[80]	Feeding & antimicrobial resistance	0-2 months	107	902	WGS	RF	Feature importance	
2024	Sizemore *et al.*^[93]^	[93]	Neurodevelopmental deficits	0-8 months	88	398	16S	Q-net model, NA	AUC	0.66-0.88
2018	Stanislawski *et al.*^[97]^	[97]	Overweight	0-2 years & 12 years	165	781	16S	RF	R-squared	0.5
2023	Vänni *et al.*^[83]^	[7,9,136-144]	Feeding & delivery mode	0-12 months		3,595	16S	RF, GB, RT	AUC	0.72-0.83
2018	Vatanen *et al.*^[58]^	[58]	T1 diabetes	3 months-15 years	783	10,913	WGS	RF	Error rates	0.45
2021	Wang *et al.*^[55]^	[1,137,145-150]	Maternal transmission	0-12 months	376	1,024	WGS	RF, GB	AUC	0.53-0.96
2023	Warner *et al.*^[95]^	[95]	Social & psychological adversity	4 months	121	121	16S/WGS	RF	AUC	0.72-0.87
2021	Xiao *et al.*^[53]^	[53,56,75,94,136,149-154]	Enterotypes	1-3 years	1,956	13,776	16S/WGS	RF	AUC	0.8
2019	Zhang *et al.*^[90]^	[90]	Atopic dermatitis	0-3 years	172	229	16S	RF	AUC	0.83

RF: Random Forest; GB: gradient boost; SVM: support vector machines; NN: neural networks; NB: naive bayes; KNN: K-nearest neighbours; EN: elastic nets; DBN: dynamic Bayesian network; LR: logistic regression; HMM: hidden Markov models; DMM: dirichlet multinomial mixtures; LASSO: lasso regression; SPLS: sparse partial least squares; EL: ensemble learning; DT: decision trees; SMOTE: synthetic minority oversampling technique; NA: network analysis; RT: randomised trees; MPPM: multilayer perceptron predictive models; AUC: under the curve.

**Table 2:** Predictive studies using microbial maturation indices to predict infant phenotypes

**Year**	**Authors**	**Dataset**	**Condition**	**Age**	**N**	**Samples**	**Data**	**Findings**
2015	Bäckhed *et al.*^[1]^	[1]	Feeding & birth mode	4-12 months	98	294	WGS	Advanced maturity in formula-fed and C-section infants
2020	Depner *et al.*^[73]^	[73]	Asthma	2-12 months	618	1,338	16S	Immaturity at 12 months associated with asthma, advanced maturity at 12 months as protective factor, *Blautia* as most age-discriminatory
2025	Fahur Bottino *et al.*^[74]^	[1,74,112,128,130,145,146,155-159]	Age	2-18 months	1,827	3,154	WGS	Uniform maturation patterns across the globe, *F. prausnitzii* & *Anaerostipes hadrus* as most age-discriminatory
2020	Galazzo *et al.*^[82]^	[82]	Birth mode, diet & atopic disorders	0-3 years	440	1453	16S	Breastfeeding as main driver of composition that delays maturation; AD related to higher early maturity and later immaturity
2023	Gao *et al.*^[12]^	[12]	Food allergy	1-12 months	323	~ 900	16S	Higher maturity at 12 months associated with lower odds of food allergy; more siblings were associated with higher maturity at 12 months
2019	Gasparrini *et al.*^[75]^	[75]	Infections	0-21 months	58	437	WGS	*F. prausnitzii* as most discriminatory of age in healthy infants; accurate MAZ scores in healthy infants; preterm infants show immaturity early on, which levels out around 12-15 months
2020	Hayden *et al.*^[110]^	[110]	Cystic fibrosis	3-12 months	207	1,157	WGS	Delayed maturation in CF infants, i.e. immaturity at 12 months
2024	Hickman *et al.*^[19]^	[19]	Health	0-5 years	984	7,211	16S	Microbial maturation highly predictable & predictive of health outcomes at 2 years & 5 years; taxa belonging to *Bifidobacterium* & *Bacteroides* were most influential in first two years
2023	Hoskinson *et al.*^[108]^	[160]	Allergy	0-5 years	589	1,238	WGS	Immaturity at 12 months related to allergies at 5 years
2019	Kamng’ona *et al.*^[161]^	[161]	Growth, inflammation	6-18 months	691	1,788	16S	Microbiota diversity and maturity were related to growth in weight from 6 to 12 months, but not to growth in length or head circumference or to growth from 12 to 18 months
2022	Kortekangas *et al.*^[109]^	[109]	Environmental enteric dysfunction	6-30 months	610	1,569	16S	Higher maturity at 18 months associated with lower EED biomarkers
2022	Lee *et al.*^[107]^	[107]	Atopic dermatitis	6-36 months	346	346	16S/WGS	*Bacteroides fragilis* and *F. prausnitzii* most discriminatory between AD and controls; advanced maturity in AD at 6 months, immaturity in AD at 12 months
2022	Lou *et al.*^[106]^	[106]	Autism spectrum disorder	11 months-19 years	1,202	1,202	16S	Immaturity at 18-30 months associated with ASD
2025	Naspolini *et al.*^[57]^	[57]	Feeding	3-9 months	525	966	WGS	Non-exclusively breastfed infants had higher maturity; EBF infants were closest to their chronological age; birth mode defines starting point: C-section/EBF had lowest maturity, Vaginal EBF the highest
2017	Pannaraj *et al.*^[104]^	[104]	Feeding	0-12 months	119	255	16S	Erysipelatotrichaceae, Bacteroidaceae and Ruminococcaceae most discriminatory between EBF and non-EBF (more abundant in non-EBF); higher maturity in infants earlier introduced to solids
2023	Robertson *et al.*^[103]^	[103]	Growth	1-18 months	335	875	WGS	Functional over compositional profiles predict growth; both functional and taxonomy predict age (*F. prausnitzii, Blautia wexlerae* & methanogenesis from acetate, L-tryptophan biosynth);
2018	Stewart *et al.*^[13]^	[13]	Age	3 months-15 years	903	12,005	16S	Immaturity in breastfed infants early on (3-30 months), but level out later (30-46 months)
2020	Stokholm *et al.*^[162]^	[9]	Asthma	0-5 years	700	1,784	16S	increased asthma risk in children born by c-section, but only if their microbial maturity at 1 year still showed c-section signature
2018	Stokholm *et al.*^[9]^	[9]	Asthma	0-5 years	690	1,696	16S	Immaturity at 12 months associated with asthma at 5 years
2014	Subramanian *et al.*^[76]^	[76]	Malnutrition	0-2 years	126	1, 585	16S	Immaturity in malnourished infants, *F. prausnitzii* most age-discriminatory
2023	Yong *et al.*^[11]^	[11]	Obesity	1-6 months	349	636	16S/ITS-2	higher early taxonomic and functional maturity (1 month) increased risk for obesity (24 months)
2022	Zhang *et al.*^[105]^	[105]	Growth	3-12 months	152	152	16S	*Actinomyces* spp most age discriminatory; immaturity in first year of life associated with failure to thrive

WGS: Whole-metagenomic shotgun sequencing; 16S: 16S rRNA gene sequencing; ITS-2: internal transcribed spacer region 2 sequencing for fungal communities; AD: atopic dermatitis; MAZ: microbiota-age-Z-score: EED: environmental enteric dysfunction; ASD: autism spectrum disorder; EBF: exclusively breastfed.

### Microbial profiles, individual taxa and community clusters as predictors of health and disease states

Studies which predict paediatric conditions based on individual or clusters of microbial taxa have been conducted for a plethora of conditions. They usually include identifying (groups of) features which are correlated or differentially abundant between respective groups, and using these features to build a predictive model, resulting in the identification and ranking of key predictive species and overall predictive performance. A prime example and one of the largest early-life microbiome ML studies to date combined a total of 13,776 global samples collected from 1 to 36 months of 1,956 infants to investigate the emergence of enterotypes during microbial succession^[53]^. The study found enterotype-specific assembly and diversity patterns, which could be stratified by geographic origin of sample, and differed in terms of birth mode, gestational age and duration of breastfeeding^[53]^.

A number of studies have undertaken the prediction of host phenotypes or early-life exposures. Le Goallec *et al.* predicted host characteristics such as breastfeeding status, sex, antibiotic use, geographic origin and mode of delivery using a variety of different ML methods^[77]^, reporting an area under the curve (AUC), i.e. a model’s ability to correctly separate cases from controls, ranging from 0.63 to 0.81. Rahman and colleagues employed Random Forest and gradient boost models to predict antimicrobial resistance genes in infants who received antibiotics *vs.* infants who did not, and found that formula-fed infants predictably harboured higher numbers of class D β-lactamase genes, enzymes which hydrolyse antibiotics and thereby confer AMR^[80]^. Similarly, other studies reported formula feeding to predict higher numbers of opportunistic pathogenic species belonging to *Staphylococcus* and *Klebsiella*, as well as an increased AMR load^[81]^.

A study conducted by Naspolini employed 966 WGS samples of 525 infants aged three to nine months and a Random Forest algorithm, reaching an accuracy ranging from 0.57 to 0.74 when predicting mode of delivery and breastfeeding status in infants. In line with previous associative studies, breastmilk feeding was found to be a major predictor of microbial composition^[58,82]^, with *Escherichia coli* and *Clostridium perfringens* discriminating strongly for exclusive breastfeeding. *Bacteroides* species were found to be highly discriminatory of mode of delivery, with vaginally-born infants displaying significantly higher relative abundances than infants^[57]^. These findings, paired with the observation that earlier timepoints (i.e. 1-2 months of age) are more predictive than later ones (3-6 months and 9-12 months), are in line with Vänni^[83]^. On a similar note, Wang employed 1,024 publicly available WGS samples and a gradient boost algorithm to predict the transmission of microbes from mother to infant, demonstrating higher similarity between related over unrelated mother-infant pairs with an accuracy of 0.69 for shared species^[55]^.

Besides discriminating host phenotypes and exposures, several early-life conditions were found to be accurately predicted when employing microbial profiles. As such, newborns with NEC can be discriminated from their healthy peers with an average accuracy of up to 90%^[84-89]^. Remarkably, models including only microbiome data had similar, if not better, performance than models containing microbiome and clinical data^[86]^; however, marked differences in performance were obtained with different ML models^[85]^. Key predictors were elevated short-chain fatty acids (SCFA) formate in NEC onset, which dissipates during recovery^[88]^, as well as taxa belonging to Enterobacteriaceae^[86,88]^, *Klebsiella*^[88,89]^, Bacillota (formerly Firmicutes) and Proteobacteria^[85,86]^. Similar success was reached when predicting atopic dermatitis, where an AUC of 83% was observed by a study conducted by Zhang^[90]^. Specifically, the authors report that lower abundances of *Bifidobacterium* are predictive of eczema in infants. With the same dataset, Ma tried five different modelling approaches and found an AUROC of up to 98% when predicting atopic dermatitis status^[91]^.

Fernández-Edreira reported an AUC of 0.8 to 0.98 when using 16S data of the DIABIMMUNE cohort, including 33 infants. In their study, *Bacteroides uniformis, Bacteroides dorei*, *Bacteroides vulgatus* and *Bacteroides thetaiotaomicron* were found to be most discriminatory between healthy controls and Type 1 Diabetes (T1D) cases, with abundances being lower in the latter^[92]^. Vatanen, however, reported a slightly better than random predictive ability with error rates of around 45% when predicting type 1 diabetes in the TEDDY cohort of infants^[58]^. Remarkably, ML approaches seem to be able to predict behavioural and neurodevelopmental conditions, including autism, with considerably high accuracy^[93]^. Loughman found microbial profiles, as opposed to clinical covariates, up to three months to be predictive of colic and problem crying in an Australian cohort^[94]^. Social and psychological adversity in four-month-old infants could be predicted with 72%-87% AUC using Random Forest algorithms, with mothers and infants showing diverse taxonomic profiles^[95]^.

Importantly, some studies also successfully predicted future disease conditions. Boutin used 545 samples obtained from 343 infants between the ages of three and twelve months to predict allergies at five years of age and found an accuracy of 81% when employing Random Forest algorithms^[96]^. Metwally longitudinally predicted food allergies at age three with an AUC of 0.69 using a deep learning framework, which outperformed various other ML algorithms^[10]^. Stanislawski used 781 samples, collected from four days to two years, of 165 Norwegian infants in a Random Forest regressor, finding gut microbiome profiles at two years of age to explain more than 50% of variation in BMI z-scores at 12 years^[97]^. Finally, Korpela *et al.* used the first stool passed by infants, as well as a follow-up sample at one year of age, to predict infant growth^[98]^. Based on marked meconial differences in taxa belonging to *Bacteroides*, *Staphylococcus* and *Lactobacillus,* their Random Forest algorithm successfully classified overweight at age three with an AUC of 0.7.

In summary, the identification of single-taxon markers offers a simple, feasible way to directly quantify and interpret microbial patterns. They potentially also allow an easy implementation and use in clinical settings, facilitated by low-cost assays such as qPCR^[99]^. Moreover, many taxa and their mechanistic links to host physiology have been identified, i.e. *Bifidobacterium* and immune development^[100]^, highlighting their potential as actionable biomarkers for monitoring early-life health. Importantly, taxa identification is constrained by the limits of microbiome data acquisition and processing, where differences between 16S and shotgun sequencing and gaps in database alignment limit the resolution, completeness and reliability of the resulting microbial profiles^[44,101]^. Moreover, focusing on individual taxa may oversimplify the highly dynamic nature of microbial ecosystems, overlooking their complex interconnections. As such, community-level classifications, such as enterotype clustering, capture broader ecological relationships and integrate multiple microbial interactions relevant to disease risk^[53]^. Nevertheless, allowing the emergence of temporally-stable clusters is limited by the aforementioned rapid shifts in early-life microbiomes, as well as highly variable microbial baselines^[1]^. Consequently, clusters differ between datasets, methodologies and geographies^[53,102]^, raising concerns about their reproducibility and deeming them currently more useful in research as opposed to clinical settings.

### Microbial maturation as a proxy for healthy developmental trajectories

Quantifying microbial succession as a process rather than utilising individual or groups of taxa provides a dynamic measure well-suited to the temporal nature of microbiome assembly. These studies use so-called microbial maturation scores, indices that assess the developmental maturity of an infant’s gut microbiome relative to healthy peers of the same chronological age. Such indices can be calculated in various ways^[19,73]^, however, the most commonly used indices are microbiota-for-age-Z-scores or MAZ-scores^[76]^. First coined by Subramanian et al. to study microbial maturity in early malnutrition^[76]^, MAZ scores are calculated by training ML models, often Random Forests, on healthy reference cohorts to predict “microbiota age” based on the abundance and presence of key microbial taxa known to change with age. The MAZ score is then derived by subtracting the child’s chronological age from their predicted microbiota age, and dividing this difference by the standard deviation of microbiota ages for that age group. Consequently, MAZ-scores are a proxy for chronological age based on microbial composition, with a value of zero denoting a typical microbiome composition for that age, while a negative and positive score indicate immaturity and advanced maturation, respectively^[76]^.

Importantly, MAZ scores have been used to quantify ‘healthy’ microbial succession patterns, with studies identifying not only highly-predictable patterns across the globe^[19,53,74]^, but also overlapping age-discriminatory compositional and functional taxa^[74-76,103]^, underscoring the possibility of defining normative trajectories of healthy microbiota development and establishing a potential clinical reference framework. These trajectories are thought to be highly influenced by early-life exposures such as family environment and, most importantly, an interactive combination of birth mode and feeding practices^[1,12,13,57,76,104]^. Bäckhed reported high maturity in formula-fed infants born via cesarean section, while Naspolini found breastfed infants born via cesarean section to have the lowest maturity and vaginally-born breastfed infants to have the highest maturity^[57]^. Exclusive breastfeeding emerged as the primary factor delaying microbial maturation^[1,13,82]^, yet maintaining maturation scores most closely aligned with chronological age^[57]^, reflecting a normative trajectory.

**Figure 2:**
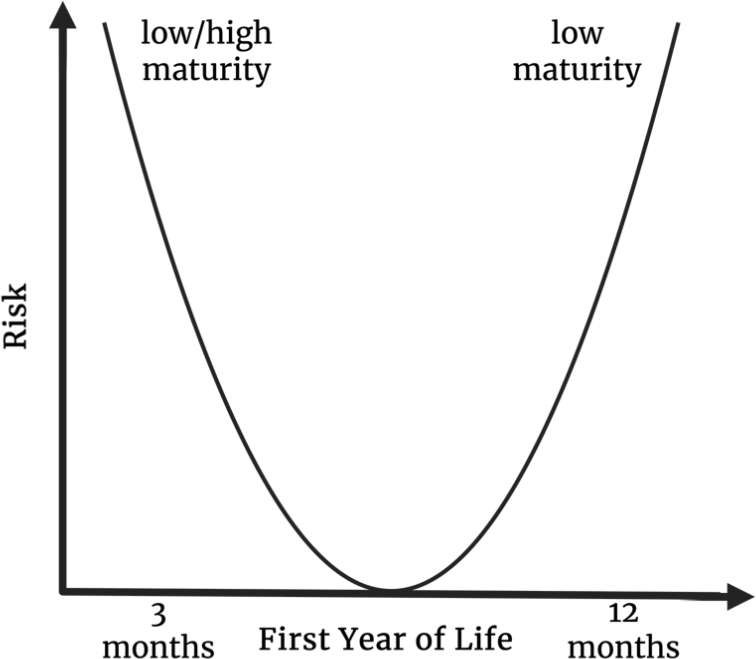
Early-life microbial maturity trajectories and implications for health; Conceptual overview summarising evidence that altered gut microbial maturation during infancy is linked to disease risk. Both unusually low or high microbial maturity in early life (birth - 3 months) and delayed maturation, characterised by low microbial maturity at 12 months, are associated with increased susceptibility to adverse health outcomes. Created in BioRender. Lavelle, A. (2026) https://BioRender.com/sxuv56p

Deviations from these expected patterns are thought to carry negative implications. Indeed, many studies indicate that both immaturity and over-maturity in early life pose risk factors for adverse health conditions, suggesting a U-shaped distribution as illustrated in Figure 2: Distribution of microbial maturity and associated risk. As such, previous research found early-life gut immaturity to be associated with growth failure^[105]^ and autism spectrum disorder^[106]^, while other studies found advanced maturity in early infancy as a risk factor for obesity^[11]^ and (food-) allergies^[12,96]^. Strikingly, the majority of studies investigating microbial maturation with health conditions report immaturity at one year of age or later to be predictive of diseases such as dermatitis^[107]^ and other atopic conditions such as asthma^[9,73,82]^ and (food) allergies^[12,96,108]^, environmental enteric dysfunction^[109]^, autism spectrum disorder^[106]^, obesity^[11]^ and impaired growth in cystic fibrosis^[110]^, as well as overall failure to thrive^[76,103,105]^. In line with these findings, a proof of concept preclinical study implanted faecal communities of varying maturity into germ-free mice and observed clear phenotypic differences, e.g. the gain of lean body mass, bone structure and metabolism^[111]^.

Taken together, studies utilising microbiota age scores attempt to define a ‘healthy’ developmental baseline; these indices act as a proxy, offering a standard against which deviations can be assessed, capturing delays or accelerated progression that may indicate risk. Unlike single-taxon or community-level measures, maturation indices are sensitive to age-related changes, reflecting the well-established role of time and developmental stage as primary drivers of infant microbiome composition^[1,13,74]^. They integrate information across multiple taxa and functions, offering a more holistic view of microbiome development, and have been shown to generalise across geographic populations^[74]^, thereby providing the greatest long-term promise as clinically relevant biomarkers that align closely with the biological reality of a developing microbiome. Nevertheless, attempting to create a benchmark of normative trajectories depends heavily on the quality of relative abundance data and representativeness of the reference cohort, demanding large and methodologically homogeneous datasets, sampled from a range of different populations^[22]^. Moreover, if used in a cross-sectional way, the metric might simplify longitudinal trajectories by assuming a linear development and neglecting baseline differences in samples. Indeed, Hickman reported not time-point specific maturity, but sequential trajectories, which take baselines as well as previous microbial communities into consideration, as predictive of later health and disease states^[19]^, underscoring the superiority of longitudinal approaches over static maturity estimates. Overall, these findings underscore the value of microbial maturation scores as a reference framework for defining healthy microbiome development and identifying deviations linked to disease risk in infancy, with the potential to guide early-life interventions in a clinical setting.

## KEY CHALLENGES IN PREDICTIVE MODELLING OF THE EARLY-LIFE MICROBIOME

However, several recurrent limitations across the above-mentioned studies emerge, tempering the strength of their conclusions. Early-life microbiome data introduce a distinct set of analytical obstacles, primarily driven by their variability^[1]^. Infant microbial communities are highly influenced by peri- and postnatal exposures, including birth mode, gestational age, antibiotic exposure, and rapidly evolving feeding practices^[13,112]^. Driving substantial inter-individual variability, these factors make it difficult for models to learn stable patterns^[23,24]^. Few of the studies adjust for a full range of these covariates^[74]^, making it difficult to disentangle microbiome-specific predictive signals from the influence of these wider determinants.

Another concern in prediction studies is overfitting due to the imbalance between high-dimensional microbial features and relatively small sample sizes^[23,48,113]^. Indeed, several key findings, including predictions of urinary tract infections^[114]^, atopic dermatitis^[91]^, Type 1 diabetes^[92]^, behavioural outcomes^[95]^, and NEC^[84]^, are derived from cohorts with fewer than 150 samples, raising questions about the representativeness of their conclusions. While sample sizes are increasing, and several recent datasets now include thousands of samples, continued expansion of well-powered, multi-cohort studies will be essential to improve generalisability and reduce model variance.

Algorithm choice is another critical factor in infant microbiome modelling, as several of the reviewed studies report differing predictive performances and findings depending on the model applied^[10,77,85,89]^. Infant microbiome data is compositional by nature, which violates the independence assumptions underlying many standard statistical models and can introduce spurious associations if not properly handled^[115]^. This challenge is further compounded by the longitudinal nature of early-life sampling, where repeated measurements vary substantially across timepoints and between case-control groups. As a result, careful preprocessing, covariate adjustment, and model selection are essential to reduce noise and improve generalisability. As such, among classical ML methods, Random Forest has consistently demonstrated strong suitability for microbiome data^[16,115-118]^. Random Forests are particularly advantageous due to their robustness to high-dimensional, sparse, and noisy data, minimal preprocessing requirements, and ability to provide intuitive measures of feature importance^[117,118]^. Gradient boosting methods, including XGBoost and LightGBM, offer strong predictive performance by sequentially optimising models, and can capture complex non-linear relationships effectively^[77,118]^. However, they are more sensitive to hyperparameter tuning, computationally intensive, and may be prone to overfitting in smaller datasets^[115]^. As a result, their performance is often dataset-dependent rather than universally superior. More complex approaches, including deep learning models such as recurrent and convolutional neural networks, may be better suited for capturing temporal dynamics in infant microbiome data^[10,24]^. However, these methods require substantially larger sample sizes and are computationally intensive, limiting their applicability in most current early-life studies. In practice, when data are limited, as is often the case in infant microbiome research, simpler ensemble methods tend to outperform deep learning approaches^[24]^. However, performance differences between ML methods are often dataset-dependent, and no single algorithm consistently dominates across all microbiome applications.

Notably, across all model classes, interpretability remains a key limitation. While Random Forests decision trees provide some degree of transparency, more complex models, including gradient boosting and neural networks, are often considered ‘black boxes’^[115,119]^. This poses a challenge for clinical translation, as identifying which taxa drive predictions is essential for biomarker discovery and potential pharmaceutical use. Post hoc interpretability tools, including SHapley Additive exPlanations (SHAP) values or permutation importance^[120,121]^, can partially address this issue, but they remain underutilised. Taken together, these considerations suggest that infant microbiome data should be handled with methods that account for compositionality, incorporate feature selection, and balance predictive performance with interpretability. Given current data constraints, ensemble tree-based methods represent robust and pragmatic choices, while more complex models may become increasingly relevant as larger, standardised datasets become available.

An additional critical and often overlooked limitation is inadequate validation. Across the studies reviewed, validation is almost exclusively restricted to internal or, in rare cases, external cohort-based evaluation. Internally validated models tend to produce overly optimistic performance estimates, with average intra-cohort performance reaching an AUC of 0.8 or higher, which typically declines substantially in external validation across independent cohorts^[16]^. This reduction in performance likely reflects both biological variability (e.g. due to differences in geography^[52]^) and technical heterogeneity, including differences in sample collection, sequencing platforms, and bioinformatic pipelines^[115]^. Notably, none of the ML-based studies reviewed here incorporated experimental validation, such as *in vivo* or *in vitro* testing of model-derived microbial signatures. Only one study extended beyond prediction by performing downstream mechanistic investigations in necrotising enterocolitis^[88]^. Casaburi and colleagues experimentally tested metabolite-driven hypotheses derived from metagenomic analyses *in vitro* and *in vivo*, demonstrating dose-dependent epithelial toxicity of candidate metabolites such as formate in rodent and human cell models. While this provides important mechanistic support for microbiome-associated disease signatures, it does not constitute validation of predictive ML models themselves, but rather offers biological plausibility for inferred microbial-metabolic associations^[88]^.

## CONCLUSIONS

In summary, current early-life ML studies provide an important foundation for future translational research. The absence of independent and experimental follow-up limits causal inference, making it difficult to determine whether previously identified microbial signatures are drivers of disease or merely correlates of underlying pathology. As larger multi-cohort datasets, longitudinal study designs, and harmonised analytical frameworks continue to emerge, microbiome-derived signals are likely to become increasingly robust, reproducible, and clinically meaningful.

As such, rather than serving as immediate diagnostic tools, current predictive models should therefore be viewed as early-stage frameworks for identifying candidate risk patterns and guiding hypothesis generation. In the long term, integrating computational predictions with experimental validation and mechanistic studies will be essential for refining these signals into biologically grounded biomarkers. With continued methodological standardisation and expanding data availability, the early-life microbiome holds strong potential to support earlier risk stratification, improved disease prediction, and ultimately more targeted preventative strategies in paediatric healthcare.

## DECLARATIONS

### Copyright

© The Author(s) 2026.

## References

[1] Bäckhed F, Roswall J, Peng Y (2015). Dynamics and stabilization of the human gut microbiome during the first year of life. Cell Host Microbe.

[2] Roswall J, Olsson LM, Kovatcheva-Datchary P (2021). Developmental trajectory of the healthy human gut microbiota during the first 5 years of life. Cell Host Microbe.

[3] Berg G, Rybakova D, Fischer D (2020). Microbiome definition re-visited: old concepts and new challenges. Microbiome.

[4] Dominguez-Bello MG, Costello EK, Contreras M (2010). Delivery mode shapes the acquisition and structure of the initial microbiota across multiple body habitats in newborns. Proc Natl Acad Sci U S A.

[5] Patangia DV, Grimaud G, O'Shea CA (2024). Early life exposure of infants to benzylpenicillin and gentamicin is associated with a persistent amplification of the gut resistome. Microbiome.

[6] Sordillo JE, Zhou Y, McGeachie MJ (2017). Factors influencing the infant gut microbiome at age 3-6 months: findings from the ethnically diverse Vitamin D Antenatal Asthma Reduction Trial (VDAART). J Allergy Clin Immunol.

[7] Levin AM, Sitarik AR, Havstad SL (2016). Joint effects of pregnancy, sociocultural, and environmental factors on early life gut microbiome structure and diversity. Sci Rep.

[8] McKeen S, Roy NC, Mullaney JA (2022). Adaptation of the infant gut microbiome during the complementary feeding transition. PLoS One.

[9] Stokholm J, Blaser MJ, Thorsen J (2018). Maturation of the gut microbiome and risk of asthma in childhood. Nat Commun.

[10] Metwally AA, Yu PS, Reiman D, Dai Y, Finn PW, Perkins DL (2019). Utilizing longitudinal microbiome taxonomic profiles to predict food allergy via Long Short-Term Memory networks. PLoS Comput Biol.

[11] Yong GJM, Porsche CE, Sitarik AR (2024). Precocious infant fecal microbiome promotes enterocyte barrier dysfuction, altered neuroendocrine signaling and associates with increased childhood obesity risk. Gut Microbes.

[12] Gao Y, Stokholm J, O'Hely M, BIS Investigator Group (2023). Gut microbiota maturity mediates the protective effect of siblings on food allergy. J Allergy Clin Immunol.

[13] Stewart CJ, Ajami NJ, O'Brien JL (2018). Temporal development of the gut microbiome in early childhood from the TEDDY study. Nature.

[14] Olaguez-Gonzalez JM, Chairez I, Breton-Deval L, Alfaro-Ponce M (2023). Machine learning algorithms applied to predict autism spectrum disorder based on gut microbiome composition. Biomedicines.

[15] Li P, Luo H, Ji B, Nielsen J (2022). Machine learning for data integration in human gut microbiome. Microb Cell Fact.

[16] Pasolli E, Truong DT, Malik F, Waldron L, Segata N (2016). Machine learning meta-analysis of large metagenomic datasets: tools and biological insights. PLoS Comput Biol.

[17] Duvallet C, Gibbons SM, Gurry T, Irizarry RA, Alm EJ (2017). Meta-analysis of gut microbiome studies identifies disease-specific and shared responses. Nat Commun.

[18] Thomas AM, Manghi P, Asnicar F (2019). Metagenomic analysis of colorectal cancer datasets identifies cross-cohort microbial diagnostic signatures and a link with choline degradation. Nat Med.

[19] Hickman B, Salonen A, Ponsero AJ (2024). Gut microbiota wellbeing index predicts overall health in a cohort of 1000 infants. Nat Commun.

[20] Morgan XC, Huttenhower C (2014). Meta'omic analytic techniques for studying the intestinal microbiome. Gastroenterology.

[21] Vandeputte D, De Commer L, Tito RY (2021). Temporal variability in quantitative human gut microbiome profiles and implications for clinical research. Nat Commun.

[22] Joos R, Boucher K, Lavelle A, Human microbiome action consortium (2025). examining the healthy human microbiome concept. Nat Rev Microbiol.

[23] Asnicar F, Thomas AM, Passerini A, Waldron L, Segata N (2024). Machine learning for microbiologists. Nat Rev Microbiol.

[24] Hernández Medina R, Kutuzova S, Nielsen KN (2022). Machine learning and deep learning applications in microbiome research. ISME Commun.

[25] Borrego-Ruiz A, Borrego JJ (2025). Early-life gut microbiome development and its potential long-term impact on health outcomes. Microbiome Res Rep.

[26] Wang S, Cui J, Jiang S (2024). Early life gut microbiota: consequences for health and opportunities for prevention. Crit Rev Food Sci Nutr.

[27] Nunez H, Nieto PA, Mars RA, Ghavami M, Sew Hoy C, Sukhum K (2025). Early life gut microbiome and its impact on childhood health and chronic conditions. Gut Microbes.

[28] Arrieta MC, Stiemsma LT, Amenyogbe N, Brown EM, Finlay B (2014). The intestinal microbiome in early life: health and disease. Front Immunol.

[29] Sarkar A, Yoo JY, Valeria Ozorio Dutra S, Morgan KH, Groer M (2021). The association between early-life gut microbiota and long-term health and diseases. J Clin Med.

[30] Wolraich M, Brown L, Brown RT, Subcommittee on Attention-Deficit/Hyperactivity Disorder, Steering Committee on Quality Improvement and Management (2011). ADHD: clinical practice guideline for the diagnosis, evaluation, and treatment of attention-deficit/hyperactivity disorder in children and adolescents. Pediatrics.

[31] Baena-Cagnani CE, Badellino HA (2011). Diagnosis of allergy and asthma in childhood. Curr Allergy Asthma Rep.

[32] Yu Z, Morrison M (2004). Improved extraction of PCR-quality community DNA from digesta and fecal samples. Biotechniques.

[33] Plauzolles A, Toumi E, Bonnet M (2022). Human stool preservation impacts taxonomic profiles in 16S metagenomics studies. Front Cell Infect Microbiol.

[34] Cardona S, Eck A, Cassellas M (2012). Storage conditions of intestinal microbiota matter in metagenomic analysis. BMC Microbiol.

[35] Gorzelak MA, Gill SK, Tasnim N, Ahmadi-Vand Z, Jay M, Gibson DL (2015). Methods for improving human gut microbiome data by reducing variability through sample processing and storage of stool. PLoS One.

[36] Choo JM, Leong LE, Rogers GB (2015). Sample storage conditions significantly influence faecal microbiome profiles. Sci Rep.

[37] Guo Y, Li SH, Kuang YS (2016). Effect of short-term room temperature storage on the microbial community in infant fecal samples. Sci Rep.

[38] Akpulu C, Lankapalli AK, Toufiq R (2025). Comparative evaluation of DNA extraction protocols for neonatal gut microbiota profiling in a resource-limited setting. The Microbe.

[39] Dorsaz S, Charretier Y, Girard M (2020). Changes in microbiota profiles after prolonged frozen storage of stool suspensions. Front Cell Infect Microbiol.

[40] Fernández-Pato A, Sinha T, Gacesa R (2024). Choice of DNA extraction method affects stool microbiome recovery and subsequent phenotypic association analyses. Sci Rep.

[41] Kazantseva J, Malv E, Kaleda A, Kallastu A, Meikas A (2021). Optimisation of sample storage and DNA extraction for human gut microbiota studies. BMC Microbiol.

[42] Hill CJ, Brown JR, Lynch DB (2016). Effect of room temperature transport vials on DNA quality and phylogenetic composition of faecal microbiota of elderly adults and infants. Microbiome.

[43] Marcos-Zambrano LJ, López-Molina VM, Bakir-Gungor B (2023). A toolbox of machine learning software to support microbiome analysis. Front Microbiol.

[44] Bars-Cortina D, Ramon E, Rius-Sansalvador B (2024). Comparison between 16S rRNA and shotgun sequencing in colorectal cancer, advanced colorectal lesions, and healthy human gut microbiota. BMC Genomics.

[45] Marcos-Zambrano LJ, Karaduzovic-Hadziabdic K, Loncar Turukalo T (2021). Applications of machine learning in human microbiome studies: a review on feature selection, biomarker identification, disease prediction and treatment. Front Microbiol.

[46] Jovel J, Patterson J, Wang W (2016). Characterization of the gut microbiome using 16S or shotgun metagenomics. Front Microbiol.

[47] Durazzi F, Sala C, Castellani G, Manfreda G, Remondini D, De Cesare A (2021). Comparison between 16S rRNA and shotgun sequencing data for the taxonomic characterization of the gut microbiota. Sci Rep.

[48] Moreno-Indias I, Lahti L, Nedyalkova M (2021). Statistical and machine learning techniques in human microbiome studies: contemporary challenges and solutions. Front Microbiol.

[49] Lin H, Peddada SD (2024). Multigroup analysis of compositions of microbiomes with covariate adjustments and repeated measures. Nat Methods.

[50] Love MI, Huber W, Anders S (2014). Moderated estimation of fold change and dispersion for RNA-seq data with DESeq2. Genome Biol.

[51] Mallick H, Rahnavard A, McIver LJ (2021). Multivariable association discovery in population-scale meta-omics studies. PLoS Comput Biol.

[52] Yatsunenko T, Rey FE, Manary MJ (2012). Human gut microbiome viewed across age and geography. Nature.

[53] Xiao L, Wang J, Zheng J, Li X, Zhao F (2021). Deterministic transition of enterotypes shapes the infant gut microbiome at an early age. Genome Biol.

[54] Kennedy KM, de Goffau MC, Perez-Muñoz ME (2023). Questioning the fetal microbiome illustrates pitfalls of low-biomass microbial studies. Nature.

[55] Wang S, Zeng S, Egan M (2021). Metagenomic analysis of mother-infant gut microbiome reveals global distinct and shared microbial signatures. Gut Microbes.

[56] Reyman M, van Houten MA, van Baarle D (2019). Impact of delivery mode-associated gut microbiota dynamics on health in the first year of life. Nat Commun.

[57] Naspolini NF, Schüroff PA, Vanzele PAR (2025). Exclusive breastfeeding is associated with the gut microbiome maturation in infants according to delivery mode. Gut Microbes.

[58] Vatanen T, Franzosa EA, Schwager R (2018). The human gut microbiome in early-onset type 1 diabetes from the TEDDY study. Nature.

[59] Fallani M, Amarri S, Uusijarvi A (2011). Determinants of the human infant intestinal microbiota after the introduction of first complementary foods in infant samples from five European centres. Microbiology (Reading).

[60] Bergström A, Skov TH, Bahl MI (2014). Establishment of intestinal microbiota during early life: a longitudinal, explorative study of a large cohort of Danish infants. Appl Environ Microbiol.

[61] Penders J, Thijs C, Vink C (2006). Factors influencing the composition of the intestinal microbiota in early infancy. Pediatrics.

[62] Azad MB, Konya T, Maughan H, CHILD Study Investigators (2013). Gut microbiota of healthy Canadian infants: profiles by mode of delivery and infant diet at 4 months. CMAJ.

[63] Li N, Yan F, Wang N (2020). Distinct gut microbiota and metabolite profiles induced by different feeding methods in healthy Chinese infants. Front Microbiol.

[64] Madan JC, Hoen AG, Lundgren SN (2016). Association of cesarean delivery and formula supplementation with the intestinal microbiome of 6-week-old infants. JAMA Pediatr.

[65] Andreas NJ, Kampmann B, Mehring Le-Doare K (2015). Human breast milk: a review on its composition and bioactivity. Early Hum Dev.

[66] Vatanen T, Plichta DR, Somani J (2019). Genomic variation and strain-specific functional adaptation in the human gut microbiome during early life. Nat Microbiol.

[67] Bode L (2015). The functional biology of human milk oligosaccharides. Early Hum Dev.

[68] Brink LR, Mercer KE, Piccolo BD (2020). Neonatal diet alters fecal microbiota and metabolome profiles at different ages in infants fed breast milk or formula. Am J Clin Nutr.

[69] Parkin K, Palmer DJ, Verhasselt V (2024). Metagenomic characterisation of the gut microbiome and effect of complementary feeding on Bifidobacterium spp. in Australian infants. Microorganisms.

[70] Thompson AL, Monteagudo-Mera A, Cadenas MB, Lampl ML, Azcarate-Peril MA (2015). Milk- and solid-feeding practices and daycare attendance are associated with differences in bacterial diversity, predominant communities, and metabolic and immune function of the infant gut microbiome. Front Cell Infect Microbiol.

[71] Tannock GW, Lawley B, Munro K (2013). Comparison of the compositions of the stool microbiotas of infants fed goat milk formula, cow milk-based formula, or breast milk. Appl Environ Microbiol.

[72] Lim ES, Zhou Y, Zhao G (2015). Early life dynamics of the human gut virome and bacterial microbiome in infants. Nat Med.

[73] Depner M, Taft DH, Kirjavainen PV, PASTURE study group (2020). Maturation of the gut microbiome during the first year of life contributes to the protective farm effect on childhood asthma. Nat Med.

[74] Fahur Bottino G, Bonham KS, Patel F (2025). Early life microbial succession in the gut follows common patterns in humans across the globe. Nat Commun.

[75] Gasparrini AJ, Wang B, Sun X (2019). Persistent metagenomic signatures of early-life hospitalization and antibiotic treatment in the infant gut microbiota and resistome. Nat Microbiol.

[76] Subramanian S, Huq S, Yatsunenko T (2014). Persistent gut microbiota immaturity in malnourished Bangladeshi children. Nature.

[77] (2020). Goallec A, Tierney BT, Luber JM, Cofer EM, Kostic AD, Patel CJ. A systematic machine learning and data type comparison yields metagenomic predictors of infant age, sex, breastfeeding, antibiotic usage, country of origin, and delivery type. PLoS Comput Biol.

[78] Tamburini S, Shen N, Wu HC, Clemente JC (2016). The microbiome in early life: implications for health outcomes. Nat Med.

[79] Stiemsma LT, Michels KB (2018). The role of the microbiome in the developmental origins of health and disease. Pediatrics.

[80] Rahman SF, Olm MR, Morowitz MJ, Banfield JF (2018). Machine learning leveraging genomes from metagenomes identifies influential antibiotic resistance genes in the infant gut microbiome. mSystems.

[81] Pärnänen KMM, Hultman J, Markkanen M (2022). Early-life formula feeding is associated with infant gut microbiota alterations and an increased antibiotic resistance load. Am J Clin Nutr.

[82] Galazzo G, van Best N, Bervoets L, GI-MDH consortium (2020). Development of the microbiota and associations with birth mode, diet, and atopic disorders in a longitudinal analysis of stool samples, collected from infancy through early childhood. Gastroenterology.

[83] Vänni P, Tejesvi MV, Paalanne N (2023). Machine-learning analysis of cross-study samples according to the gut microbiome in 12 infant cohorts. mSystems.

[84] Dobbler PT, Procianoy RS, Mai V (2017). Low microbial diversity and abnormal microbial succession is associated with necrotizing enterocolitis in preterm infants. Front Microbiol.

[85] Hooven TA, Lin AYC, Salleb-Aouissi A (2020). Multiple instance learning for predicting necrotizing enterocolitis in premature infants using microbiome data. Proc ACM Conf Health Inference Learn (2020).

[86] Lin YC, Salleb-Aouissi A, Hooven TA (2022). Interpretable prediction of necrotizing enterocolitis from machine learning analysis of premature infant stool microbiota. BMC Bioinformatics.

[87] Masi AC, Embleton ND, Lamb CA (2021). Human milk oligosaccharide DSLNT and gut microbiome in preterm infants predicts necrotising enterocolitis. Gut.

[88] Casaburi G, Wei J, Kazi S (2022). Metabolic model of necrotizing enterocolitis in the premature newborn gut resulting from enteric dysbiosis. Front Pediatr.

[89] Olm MR, Bhattacharya N, Crits-Christoph A (2019). Necrotizing enterocolitis is preceded by increased gut bacterial replication, Klebsiella, and fimbriae-encoding bacteria. Sci Adv.

[90] Zhang Y, Jin S, Wang J (2019). Variations in early gut microbiome are associated with childhood eczema. FEMS Microbiol Lett.

[91] Ma J, Fang Y, Li S (2025). Interpretable machine learning algorithms reveal gut microbiome features associated with atopic dermatitis. Front Immunol.

[92] Fernández-Edreira D, Liñares-Blanco J, Fernandez-Lozano C (2021). Machine learning analysis of the human infant gut microbiome identifies influential species in type 1 diabetes. Expert Syst Appl.

[93] Sizemore N, Oliphant K, Zheng R, Martin CR, Claud EC, Chattopadhyay I (2024). A digital twin of the infant microbiome to predict neurodevelopmental deficits. Sci Adv.

[94] Loughman A, Quinn T, Nation ML (2021). Infant microbiota in colic: predictive associations with problem crying and subsequent child behavior. J Dev Orig Health Dis.

[95] Warner BB, Rosa BA, Ndao IM (2023). Social and psychological adversity are associated with distinct mother and infant gut microbiome variations. Nat Commun.

[96] Boutin RCT, Sbihi H, McLaughlin RJ (2021). Composition and associations of the infant gut fungal microbiota with environmental factors and childhood allergic outcomes. mBio.

[97] Stanislawski MA, Dabelea D, Wagner BD (2018). Gut microbiota in the first 2 years of life and the association with body mass index at age 12 in a Norwegian birth cohort. mBio.

[98] Korpela K, Renko M, Vänni P (2020). Microbiome of the first stool and overweight at age 3 years: a prospective cohort study. Pediatr Obes.

[99] Rodriguez J, Hassani Z, Alves Costa Silva C, Human Microbiome Action consortium (2025). State of the art and the future of microbiome-based biomarkers: a multidisciplinary Delphi consensus. Lancet Microbe.

[100] Henrick BM, Rodriguez L, Lakshmikanth T (2021). Bifidobacteria-mediated immune system imprinting early in life. Cell.

[101] Anwar S, Lamaudiere M, Hassall J (2025). DNA reference reagents isolate biases in microbiome profiling: a global multi-lab study. mSystems.

[102] Koren O, Knights D, Gonzalez A (2013). A guide to enterotypes across the human body: meta-analysis of microbial community structures in human microbiome datasets. PLoS Comput Biol.

[103] Robertson RC, Edens TJ, Carr L (2023). The gut microbiome and early-life growth in a population with high prevalence of stunting. Nat Commun.

[104] Pannaraj PS, Li F, Cerini C (2017). Association between breast milk bacterial communities and establishment and development of the infant gut microbiome. JAMA Pediatr.

[105] Zhang M, Miao D, Ma Q (2022). Underdevelopment of gut microbiota in failure to thrive infants of up to 12 months of age. Front Cell Infect Microbiol.

[106] Lou M, Cao A, Jin C (2022). Deviated and early unsustainable stunted development of gut microbiota in children with autism spectrum disorder. Gut.

[107] Lee MJ, Park YM, Kim B (2022). Disordered development of gut microbiome interferes with the establishment of the gut ecosystem during early childhood with atopic dermatitis. Gut Microbes.

[108] Hoskinson C, Dai DLY, Del Bel KL (2023). Delayed gut microbiota maturation in the first year of life is a hallmark of pediatric allergic disease. Nat Commun.

[109] Kortekangas E, Fan YM, Chaima D (2022). Associations between gut microbiota and intestinal inflammation, permeability and damage in young Malawian children. J Trop Pediatr.

[110] Hayden HS, Eng A, Pope CE (2020). Fecal dysbiosis in infants with cystic fibrosis is associated with early linear growth failure. Nat Med.

[111] Blanton LV, Charbonneau MR, Salih T (2016). Gut bacteria that prevent growth impairments transmitted by microbiota from malnourished children. Science (1979).

[112] Yassour M, Vatanen T, Siljander H, DIABIMMUNE Study Group (2016). Natural history of the infant gut microbiome and impact of antibiotic treatment on bacterial strain diversity and stability. Sci Transl Med.

[113] Dudek NK, Chakhvadze M, Kobakhidze S, Kantidze O, Gankin Y (2024). Supervised machine learning for microbiomics: Bridging the gap between current and best practices. Mach Learn Appl.

[114] Piteková B, Hric I, Zieg J (2025). The gut microbiome and metabolome in children with a first febrile urinary tract infection: a pilot study. Pediatr Nephrol.

[115] Papoutsoglou G, Tarazona S, Lopes MB (2023). Machine learning approaches in microbiome research: challenges and best practices. Front Microbiol.

[116] Topçuoğlu BD, Lesniak NA, Ruffin MT 4th, Wiens J, Schloss PD (2020). A framework for effective application of machine learning to microbiome-based classification problems. mBio.

[117] Statnikov A, Henaff M, Narendra V (2013). A comprehensive evaluation of multicategory classification methods for microbiomic data. Microbiome.

[118] Li P, Li M, Chen WH (2025). Best practices for developing microbiome-based disease diagnostic classifiers through machine learning. Gut Microbes.

[119] Guidotti R, Monreale A, Ruggieri S, Turini F, Giannotti F, Pedreschi D (2019). A survey of methods for explaining black box models. ACM Comput Surv.

[120] Lundberg SM, Allen PG, Lee SI (2017). A unified approach to interpreting model predictions. Adv Neural Inf Process Syst.

[121] Altmann A, Toloşi L, Sander O, Lengauer T (2010). Permutation importance: a corrected feature importance measure. Bioinformatics.

[122] Raveh-Sadka T, Firek B, Sharon I (2016). Evidence for persistent and shared bacterial strains against a background of largely unique gut colonization in hospitalized premature infants. ISME J.

[123] Rose G, Shaw AG, Sim K (2017). Antibiotic resistance potential of the healthy preterm infant gut microbiome. PeerJ.

[124] Gibson MK, Wang B, Ahmadi S (2016). Developmental dynamics of the preterm infant gut microbiota and antibiotic resistome. Nat Microbiol.

[125] Tisza MJ, Buck CB (2021). A catalog of tens of thousands of viruses from human metagenomes reveals hidden associations with chronic diseases. Proc Natl Acad Sci U S A.

[126] Brooks B, Olm MR, Firek BA (2017). Strain-resolved analysis of hospital rooms and infants reveals overlap between the human and room microbiome. Nat Commun.

[127] Chen W, Zhang P, Zhang X (2024). Machine learning-causal inference based on multi-omics data reveals the association of altered gut bacteria and bile acid metabolism with neonatal jaundice. Gut Microbes.

[128] Kostic AD, Gevers D, Siljander H, DIABIMMUNE Study Group (2015). The dynamics of the human infant gut microbiome in development and in progression toward type 1 diabetes. Cell Host Microbe.

[129] Warner BB, Deych E, Zhou Y (2016). Gut bacteria dysbiosis and necrotising enterocolitis in very low birthweight infants: a prospective case-control study. Lancet.

[130] Vatanen T, Kostic AD, d’Hennezel E, DIABIMMUNE Study Group (2016). Variation in microbiome LPS immunogenicity contributes to autoimmunity in humans. Cell.

[131] Lugo-Martinez J, Xu S, Levesque J (2022). Integrating longitudinal clinical and microbiome data to predict growth faltering in preterm infants. J Biomed Inform.

[132] Martin VM, Virkud YV, Dahan E (2022). Longitudinal disease-associated gut microbiome differences in infants with food protein-induced allergic proctocolitis. Microbiome.

[133] McGeachie MJ, Sordillo JE, Gibson T (2016). Longitudinal prediction of the infant gut microbiome with dynamic Bayesian networks. Sci Rep.

[134] La Rosa PS, Warner BB, Zhou Y (2014). Patterned progression of bacterial populations in the premature infant gut. Proc Natl Acad Sci U S A.

[135] Nguyen QP, Karagas MR, Madan JC (2021). Associations between the gut microbiome and metabolome in early life. BMC Microbiol.

[136] Hill CJ, Lynch DB, Murphy K (2017). Evolution of gut microbiota composition from birth to 24 weeks in the INFANTMET cohort. Microbiome.

[137] Chu DM, Ma J, Prince AL, Antony KM, Seferovic MD, Aagaard KM (2017). Maturation of the infant microbiome community structure and function across multiple body sites and in relation to mode of delivery. Nat Med.

[138] Laursen MF, Andersen LB, Michaelsen KF (2016). Infant gut microbiota development is driven by transition to family foods independent of maternal obesity. mSphere.

[139] Jokela R, Korpela K, Jian C (2022). Quantitative insights into effects of intrapartum antibiotics and birth mode on infant gut microbiota in relation to well-being during the first year of life. Gut Microbes.

[140] Lundgren SN, Madan JC, Emond JA (2018). Maternal diet during pregnancy is related with the infant stool microbiome in a delivery mode-dependent manner. Microbiome.

[141] Iszatt N, Janssen S, Lenters V (2019). Environmental toxicants in breast milk of Norwegian mothers and gut bacteria composition and metabolites in their infants at 1 month. Microbiome.

[142] Tapiainen T, Koivusaari P, Brinkac L (2019). Impact of intrapartum and postnatal antibiotics on the gut microbiome and emergence of antimicrobial resistance in infants. Sci Rep.

[143] Russell JT, Roesch LFW, Ördberg M (2019). Genetic risk for autoimmunity is associated with distinct changes in the human gut microbiome. Nat Commun.

[144] Robinson A, Fiechtner L, Roche B (2017). Association of maternal gestational weight gain with the infant fecal microbiota. J Pediatr Gastroenterol Nutr.

[145] Asnicar F, Manara S, Zolfo M (2017). Studying vertical microbiome transmission from mothers to infants by strain-level metagenomic profiling. mSystems.

[146] Shao Y, Forster SC, Tsaliki E (2019). Stunted microbiota and opportunistic pathogen colonization in caesarean-section birth. Nature.

[147] Pärnänen K, Karkman A, Hultman J (2018). Maternal gut and breast milk microbiota affect infant gut antibiotic resistome and mobile genetic elements. Nat Commun.

[148] Yassour M, Jason E, Hogstrom LJ (2018). Strain-level analysis of mother-to-child bacterial transmission during the first few months of life. Cell Host Microbe.

[149] Wampach L, Heintz-Buschart A, Fritz JV (2018). Birth mode is associated with earliest strain-conferred gut microbiome functions and immunostimulatory potential. Nat Commun.

[150] Ferretti P, Pasolli E, Tett A (2018). Mother-to-infant microbial transmission from different body sites shapes the developing infant gut microbiome. Cell Host Microbe.

[151] Gregory KE, Samuel BS, Houghteling P (2016). Influence of maternal breast milk ingestion on acquisition of the intestinal microbiome in preterm infants. Microbiome.

[152] Matsuki T, Yahagi K, Mori H (2016). A key genetic factor for fucosyllactose utilization affects infant gut microbiota development. Nat Commun.

[153] Raman AS, Gehrig JL, Venkatesh S (2019). A sparse covarying unit that describes healthy and impaired human gut microbiota development. Science.

[154] Tauchi H, Yahagi K, Yamauchi T (2019). Gut microbiota development of preterm infants hospitalised in intensive care units. Benef Microbes.

[155] Hemmingway A, Fisher D, Berkery T, Dempsey E, Murray DM, Kiely ME (2020). A detailed exploration of early infant milk feeding in a prospective birth cohort study in Ireland: combination feeding of breast milk and infant formula and early breast-feeding cessation. Br J Nutr.

[156] Portlock T, Shama T, Kakon SH (2025). Interconnected pathways link faecal microbiota plasma lipids and brain activity to childhood malnutrition related cognition. Nat Commun.

[157] Fatori D, Shephard E, Benette D (2025). Identifying biomarkers and trajectories of executive functions and language development in the first 3 years of life: design, methods, and findings of the Germina cohort study. Dev Psychopathol.

[158] Bonham KS, Fahur Bottino G, McCann SH, RESONANCE Consortium (2023). Gut-resident microorganisms and their genes are associated with cognition and neuroanatomy in children. Sci Adv.

[159] Pehrsson EC, Tsukayama P, Patel S (2016). Interconnected microbiomes and resistomes in low-income human habitats. Nature.

[160] Moraes TJ, Lefebvre DL, Chooniedass R, CHILD Study Investigators (2015). The Canadian healthy infant longitudinal development birth cohort study: biological samples and biobanking. Paediatr Perinat Epidemiol.

[161] Kamng’ona AW, Young R, Arnold CD (2019). The association of gut microbiota characteristics in Malawian infants with growth and inflammation. Sci Rep.

[162] Stokholm J, Thorsen J, Blaser MJ (2020). Delivery mode and gut microbial changes correlate with an increased risk of childhood asthma. Sci Transl Med.

